# AMTB, a TRPM8 antagonist, suppresses growth and metastasis of osteosarcoma through repressing the TGFβ signaling pathway

**DOI:** 10.1038/s41419-022-04744-6

**Published:** 2022-03-31

**Authors:** Yujie Liu, Ao Leng, Lin Li, Bo Yang, Shihui Shen, Hui Chen, Enhao Zhu, Qiyue Xu, Xiaoyu Ma, Peilin Shi, Yun Liu, Tielong Liu, Lei Li, Kun Li, Dan Zhang, Jianru Xiao

**Affiliations:** 1 Department of Orthorpedics,Department of Orthopedic Oncology and Spine Tumor Center, Changzheng Hospital, Navy Medical University, Shanghai, China; 2 Shanghai Key Laboratory of Regulatory Biology, Institute of Biomedical Sciences, School of Life Sciences, East China Normal University, Shanghai, China

**Keywords:** Biochemistry, Bone cancer

## Abstract

Since its first identification in prostate cancers and prostate tissues, transient receptor potential melastatin-subfamily member 8 (TRPM8) is subsequently found to be overexpressed in a wide range of cancers and is shown to be implicated in tumorigenesis and tumor progression. Here, we used N-(3-aminopropyl)-2-[(3-methylphenyl) methoxy] -N-(2-thienylmethyl) benzamide hydrochloride (AMTB), a specific TRPM8 antagonist, to explore its antitumoral effect on osteosarcoma. We find that AMTB suppresses osteosarcoma cell proliferation, metastasis and induces cellular apoptosis. Xenograft model in nude mice experiments also define that AMTB can increase the sensitivity of tumor cells to cisplatin, the cytotoxic chemotherapeutic regimens in treating osteosarcoma. Molecularly, AMTB specifically antagonizes TRPM8 which is upregulated in osteosarcoma and its expression level in osteosarcoma tissues is negatively related to patients’ prognosis. Finally, RNA sequencing analysis was performed to explore the mechanism underlying the antitumoral effect of AMTB on osteosarcoma cells and the results prove that AMTB suppresses the Transforming Growth Factor β (TGFβ) signaling pathway. Our study provides evidence that TRPM8 could be a potential therapeutic target and AMTB can suppress growth and metastasis of osteosarcoma cells through repressing the TGFβ signaling pathway and increase the sensitivity of tumor cells to cisplatin.

## Introduction

Osteosarcoma is the most common primary malignant bone tumor in children and adolescents, with a second peak in incidence in those over the age of 60 [1]. Surgery, so far, remains the mainstay of treatment in resectable osteosarcoma [2]. In the meanwhile, the addition of multiagent chemotherapy adopting methotrexate, doxorubicin, and cisplatin (MAP) has significantly improved the outcomes of patients with localized lesions, with the 5-year survival rate approaching 60–70% [3]. However, since the mid-1980s, little progress has been achieved, and the outcomes for patients with relapsed or metastatic disease remain dismal [4]. Despite increasing knowledge of its molecular etiology, osteosarcoma-specific antigens have been difficult to identify, and the search for common molecular therapeutic targets has been disappointing [5]. Ongoing studies concerning immunotherapies may hold the promise of future advances, but currently osteosarcoma remains an intractable challenge for clinical physicians [6].

TRPM8 is a non-selective, voltage gated, and Ca^2+^ permeable cation channel, belonging to the superfamily of TRP proteins [7]. In human tissues, TRPM8 is selectively expressed in the male urogenital system, liver, pancreas, stomach and peripheral sensory neurons [7, 8]. Under physiological conditions, TRPM8 can be activated by innocuous cooling or cooling agents such as menthol and icilin, and is implicated in thermosensation and nociception [9]. Recent work has revealed that TRPM8 also contributes to the development of platinum-induced neuropathy, where TRPM8 is observed to be upregulated after treatment with platinum [10]. Additionally, accumulating evidence indicates that TRPM8 is aberrantly upregulated in various cancers, and plays a promoting role in tumorigenesis and tumor progression [11, 12]. AMTB, a specific TRPM8 antagonist, was proved to have an antinociceptive role against hyperalgesia induced by inflammation or cold stimuli [13–15]. Relevant to the present study, AMTB was shown to have an antiproliferative role in Caco-2 cells (colorectal carcinoma) [16] and can suppress the migration of LNCaP, PC3, and DU145 cells (prostate cancer cells) [17]. Nevertheless, the systematic function and the underlying mechanism of AMTB against tumor cells has not been fully recognized. Herein, we investigated the effect of AMTB on osteosarcoma.

TGFβ family is a class of cytokines that controls a variety of biological processes, including proliferation, differentiation, morphogenesis, and tissue homeostasis [18]. Three different isoforms of TGFβ have been identified in mammals, including TGFβ1, TGFβ2, and TGFβ3. Once activated, TGFβ dimers can bind with the serine/threonine kinase receptor cell surface complexes (TGFBR1 and TGFRR2), resulting in the phosphorylation of the type 1 receptor by the type 2 receptor. The phosphorylated type 1 receptor then recruits and phosphorylates receptor-regulated Smads (R-Smads). Activated R-Smads then form heteromeric complex with the common partner Smad4, and translocate into the nucleus, where they associate with other DNA-binding transcription factors to achieve high affinity and selectivity for target promoters [19]. In addition to the canonical Smads signaling, TGFβ is also known to regulate non-Smads pathways, including ERK, p38 MAPK, JNK, PI3k-Akt, and small GTPase [20].

Given the comprehensive biological function of TGFβ signaling, distortion of such signaling may lead to severe diseases. Evidence has shown that perturbations of TGFβ signaling are central to tumorigenesis and tumor progression [21]. In most epithelial and hematopoietic carcinoma, TGFβ signaling acts as a tumor suppressor during early stages, but contrarily promotes metastasis as the tumor progresses [22]. Previous studies have revealed that TGFβ stimulation can not only inhibit cell cycle progression in the G1 phase through the induction of cyclin-dependent kinase inhibitors and p21, but also represses the expression of MYC, which plays a crucial role during cell proliferation [23, 24]. However, in contrast with carcinoma, TGFβ fails to inhibit mesenchymal cell proliferation, and promotes the progression of osteosarcoma through the induction of platelet-derived growth factor (PDGF) [25]. In addition, aberrant upregulation of TGFβ in the tumor microenvironment is implicated in angiogenesis and bone remodeling in a way that favors osteosarcoma progression and metastasis [26]. Herein, we demonstrated that AMTB can impede tumor progression through repressing the TGFβ signaling in osteosarcoma.

## Materials and methods

### Reagents

AMTB was purchased from Merck KGaA company (#SML0103). Cisplatin was purchased from Sigma-Aldrich company (#61825-94-3). The antibody to TRPM8 was bought from Abcam (#ab3243). The antibodies to Caspase-3 (#9662), PARP (#9542), Smad2/Smad3 (#8685) and Phospho-Smad2 (Ser465/467)/Smad3 (Ser423/425) (#8828) were bought from Cell Signaling Technology, Inc. The antibody to β-Actin was bought from Medical & Biological Laboratories Co., LTD. (#M177-3).

### Tissue samples

All tumor tissues and adjacent non-tumoral tissues were obtained from patients surgically treated at the Department of Orthopedic Oncology at Shanghai Changzheng Hospital. The histologic diagnosis of osteosarcoma was confirmed by two independent pathologists after surgical resection. All samples were immediately snap-frozen in liquid nitrogen after surgery, and stored in liquid nitrogen until further use. Our work has been carried out in accordance with The Code of Ethics of the World Medical Association (Declaration of Helsinki). Our study was approved by the Ethics Committees of Shanghai Changzheng Hospital with written informed consents obtained from all patients or their legal guardians.

### Immunohistochemistry analysis

Paraffin-embedded tumor tissues and adjacent non-tumoral tissues were sectioned and immunohistochemically stained for TRPM8 (1:100, Abcam, MA, USA) using the Rabbit Immunohistochemistry (IHC) kit bought from Shanghai Maokang Biotechnology Co., Ltd.

Tissues were embedded in paraffin and then cut into different sections (4–5 mm thick). Immunohistochemical staining was scored according to the following standards: staining intensity (I) was classified as 0 (lack of staining), 1 (mild staining), 2 (moderate staining) or 3 (strong staining); staining percentage (P) was designated as 1 (<25%), 2 (25%–50%), 3 (51%–75%), or 4 (>75%). For each section, the semiquantitative score was calculated by multiplying I and P (which ranged from 0 to 12). Score 0–3 was as not significant (negative), 4–8 as weakly positive and 9–12 as strongly positive. In the analysis, low expression meant negative or weakly positive, high expression meant strongly positive. The log-rank test was performed to assess statistical significance.

### Cell culture

Mesenchymal stem cells (MSC) and osteosarcoma cell lines (U2OS, 143B, MG-63, HOS) were obtained from the American Type Culture Collection. Each cell line was propagated and maintained according to the instructions of the corresponding provider. The cell lines U2OS, 143B, MG-63, and HOS were cultured in Dulbecco’s modified Eagle’s medium (ThermoFisher Biochemical Products, Beijing, China) while MSC were cultured in Human Mesenchymal Stem Cell Growth Medium (Cyagen Biosciences, Santa Clara, USA), all supplemented with 10% fetal bovine serum (Epizyme, Massachusetts, USA), 100 IU/ml penicillin G sodium and 100 μg/ml streptomycin sulfate (Basalmedia, Shanghai, China). Cells were incubated in a humidified incubator containing 5% CO_2_ at 37 °C.

### MTS assay

Cell viability was measured using the CellTiter 96® AQueous One Solution Reagent (Promega, Wisconsin, USA). In brief, cells were seeded into 96-well plates at a density of 1500 cells/well. AMTB of different concentrations (0 μM, 5 μM, 10 μM) were added to the medium after 24 h cultivation. Cells were then cultivated at 37 °C for different time periods (0 h, 24 h, 48 h, 72 h, 96 h). Upon measurement, 20 μl of the CellTiter 96® AQueous One Solution Reagent mixed with 100 μl serum-free DMEM medium was added to each well and incubated for 60 min. The optical density (OD) values of the supernatant were then measured at 490 nm and compared within different groups. Each experiment was independently repeated for three times.

### Colony formation assay

For colony formation assay, cells were trypsinized and equally seeded into 6-well plates at a density of 1000 cells/well. AMTB of different concentrations (0 μM, 2.5 μM, 5.0 μM) were added accordingly to the medium after 24 h cultivation. After further cultivation for 7 days, the cells were fixed with 4% polyoxymethylene and stained with 0.1% crystal violet. Images of stained colonies were taken, and the numbers of the colonies were recorded and compared within different groups. Each experiment was independently repeated for three times.

### Wound-healing assay

After cells were cultured to a confluence around 90%, a small area was disrupted by scratching the monolayer with a 200 μl plastic pipette tip. Afterwards, cells were cultured for 24 h with low serum medium (1%) containing different concentrations of AMTB (0 μM, 5 μM, 10 μM). Images were taken right after the scratching and after 24 h cultivation. The migration rate, estimated by wound area right after scratching divided by wound area after 24 h cultivation, was calculated and compared within different groups. Each experiment was independently repeated for three times.

### Transwell assay

Transwell assay was carried out using transwell inserts with 6.5-mm-diameter and 5.0 µm Pore Polycarbonate Membrane (Corning Inc., Maine, USA). Serum-free medium containing 1.5 × 10^5^ U2OS or 143B cells complemented with different concentrations (0 μM, 5 μM, 10 μM) of AMTB were added to the inside compartments of the transwell inserts, whereas the lower chambers were filled with full medium containing 10% FBS. After incubation for 24 h, the lower surface of the inserts were washed twice with cold PBS, fixed with 4% polyoxymethylene and stained with 0.1% crystal violet. Each experiment was independently repeated for three times.

### Flow cytometry

After treating cells with different concentrations of AMTB (0 μM, 10 μM, 20 μM, 30 μM) for 24 h. Cell apoptosis was detected using Annexin V-FITC apoptosis detection kit (Boster Biological Technology Co., Ltd., California, USA) in accordance with the manufacturer’s instructions. Specimens were analyzed with BD FACS Calibur (Beckman Coulter, CA, USA) and the apoptotic rate was determined by the FlowJo software (Tree Star Inc., Ashland, USA). Each experiment was independently repeated for three times.

### Western blotting analysis

Cells were resuspended in SDS sample buffer (Invitrogen, California, USA) and resolved in 12% SDS gels. Proteins were transferred to nitrocellulose membranes and then immunoblotted with primary antibodies specific for β-actin, TRPM8, caspase-3, PARP, Smad2/Smad3 and phospho- Smad2/Smad3 overnight at 4 °C. After incubation with fluorescent-labeled secondary antibodies (1:5000 dilution for M680 and 1:10,000 dilution for R800) for an hour, signals for proteins were visualized with the LI-COR Odyssey Infrared Imaging System.

### RNA isolation and RT-qPCR

Total RNA was extracted from cells using Total RNA Extraction Reagent (Vazyme Biotech, Nanjing, China) and reversely transcribed into cDNA with HiScript II Reverse Transcriptase (Vazyme, Nanjing, China) according to the manufacturer’s protocol. Quantitative real time-PCR (RT-qPCR) was conducted with Applied Biosystems 7900HT (Applied Biosystems). In all, 18 s was used as the internal control to normalize the variability in expression levels. The primer sequence involved are listed as follows: (Biosune Biotech, Shanghai, China) 5′-GGACACGGACAGGATTGACA-3′ (forward) and 3′-GACATCTAAGGGCATCACAG-5′ (reverse) for 18 s, 5′-CCATCCCGCCCACTTTCTAC-3′ (forward) and 3′-AGCTCAATCCGTTGTTCAGGC-5′ (reverse) for TGFβ2, 5′-ACCGACTGGAAGACACGTTTG-3′ (forward) and 3′-CCAGGTCAGCTTCGCAAGG-5′ (reverse) for CTGF, 5′-AGACTCCGCATCGCAAAGG-3′ (forward) and 3′-TCACCACGTTGTTGTCAAGGG-5′ (reverse) for THBS1, 5′-TGTCCGTCAGAACCCATGC-3′ (forward) and 3′-AAAGTCGAAGTTCCATCGCTC-5′ (reverse) for CDKN1A. The 2-^ΔΔCt^ method was used to determine the relative quantitation of mRNA expression. Reactions were run in triplicate in three independent experiments.

### RNA sequencing

Total RNA was extracted by Trizol regent, and the quality was assessed by Bioanalyzer 2100 system (Agilent Technologies, CA, USA). In total, 2 µg total RNA per sample was used for RNA sequencing by rRNA depleting method. The data of per sample was obtained on Illumina NovaSeq platform for following analysis.

### Preclinical experimental model of osteosarcoma

In total, 30 female BALB/c nude mice were purchased from Shanghai Slac Laboratory Animal Co. Ltd. and raised in specific pathogen-free environment until six-week-old. 5 × 10^6^ U2OS cells were suspended per 100 μl chilled PBS and subcutaneously injected into the back of each mouse. Mice were then raised and observed on a daily basis until the tumors reached a volume ~200 mm^3^. 24 mice bearing similar size of tumors were then randomly allocated into four treatment groups: (1) 100 μl 5% glucose + 100 μl PBS, (2) 100 μl 5% glucose + AMTB (6 mg/Kg) resolved in 100 μl PBS, (3) Cisplatin (6 mg/Kg) resolved in 100 μl 5% glucose + 100 μl PBS, and (4) Cisplatin (4 mg/Kg) dissolved in 100 μl 5% glucose + AMTB (6 mg/Kg) resolved in 100 μl PBS. Mice in different groups were treated on a once every two days basis while tumor sizes were concomitantly documented by the formula: tumor volume = 0.5 × L × W^2^. After a total of 3 weeks treatment, mice were sacrificed and the tumors were detached, weighted, and fixed using 10% formalin for additional examinations. All experiments were conducted in compliance with the National Institutes of Health Animal Use Guidelines and authorized by the Laboratory Animal Center of East China Normal University.

### Statistical analysis

GraphPad Prism 7.0 (CA, USA) and IBM SPSS Statistics 21.0 (IL, USA) were used for data analysis. Obtained data were presented as mean ± standard deviation (SD). One-way ANOVA was applied for analysis between different groups, while the Kaplan–Meier method and log rank test were employed for comparison of patients’ overall survival with different TRPM8 expression level. *P* values are indicated in the figures by asterisks near the corresponding column or symbol. *p < 0.05; **p < 0.01; ***p < 0.005.

## Results

### TRPM8 is upregulated in osteosarcoma and may serve as a valuable prognostic marker

**Fig. 1:**
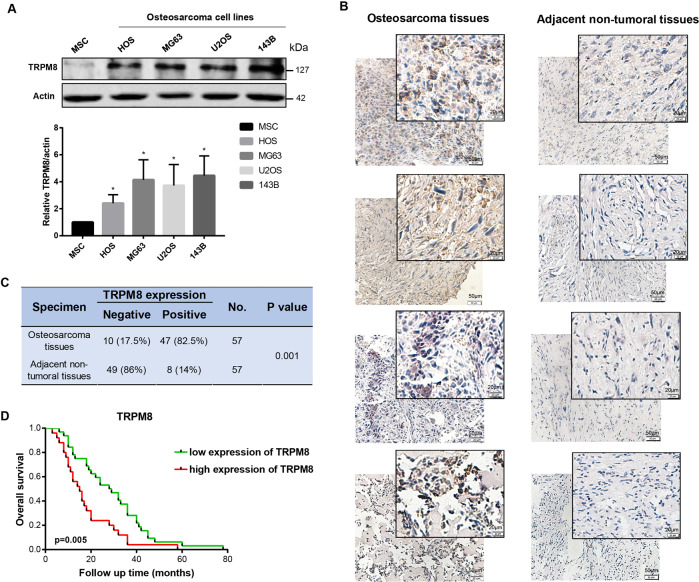
TRPM8 is upregulated and correlated with a poor prognosis in osteosarcoma. **A** TRPM8 expression in osteosarcoma cells and MSC as determined by western blot (top) . Western blot is representative of five independent western blot with different lysates. TRPM8 levels for each compound was normalized to MSC cell values (bottom) . The data was compared by one-way ANOVA with Tukey’s multiple comparison test and shown as mean ± s.d. *P < 0.05. **B**, **C** Expression of TRPM8 in 57 osteosarcoma samples and paired adjacent non-tumoral samples as determined by IHC staining. **D** Kaplan–Meier survival rates for osteosarcoma patients with non/weak staining or moderate/strong staining of TRPM8.

Expression level of TRPM8 was examined in 4 osteosarcoma cell lines (143B, U2OS, MG63, and HOS) and human mesenchymal stem cells (hMSC) using Western blot assay. Results indicate that TRPM8 is overexpressed in all four osteosarcoma cell lines, with at least two-fold increase as compared to that in MSC (Fig. 1A). Next, we detected the expression of TRPM8 in 57 human osteosarcoma samples and paired adjacent non-tumoral samples (Fig. 1B). In compliance with previous observations, 47/57 (82.5%) osteosarcoma samples and only 8/57 (14%) adjacent non-tumoral samples were tested positive for TRPM8, verifying a differential expression of TRPM8 between osteosarcoma and adjacent non-tumoral tissues (*p* = 0.001) (Fig. 5C). Furthermore, patients diagnosed with osteosarcoma were subdivided into non/weak staining group or moderate/strong staining group based on their IHC presentations. The Kaplan–Meier method was adopted to estimate the overall survival (OS) of patients in different groups, and the log-rank test was employed for comparison of the OS. OS for patients with non/weak staining of TRPM8 was 40.0 ± 5.8 months, while the OS for patients with moderate/strong staining of TRPM8 was 21.6 ± 2.6 months, indicating a negative relationship between TRPM8 expression and patients’ prognosis (*p* = 0.005) (Fig. 5D). Taken together, TRPM8 is upregulated in both osteosarcoma cell lines and osteosarcoma samples, and such upregulation has an indicative role in predicting patients’ prognosis.

### AMTB suppresses osteosarcoma cell proliferation and migration in vitro

**Fig. 2:**
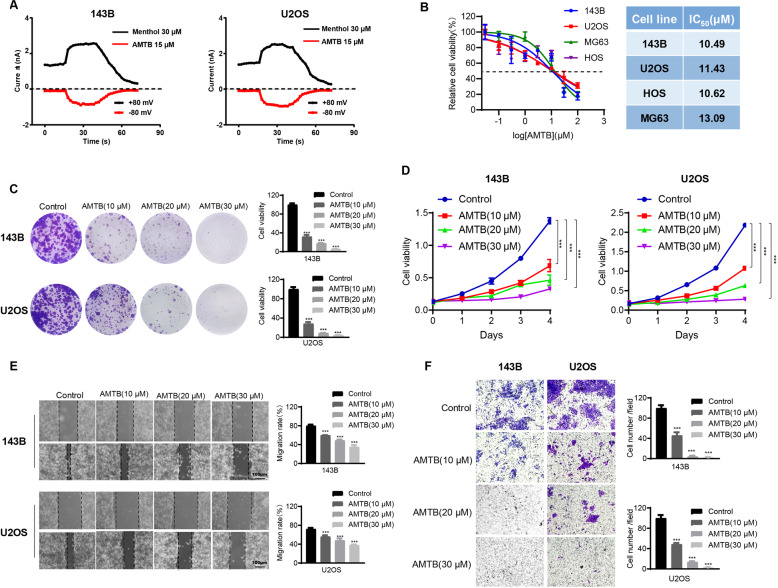
AMTB suppresses osteosarcoma cell proliferation and migration in vitro. **A** Time course of menthol-induced whole-cell currents in 143B and U2OS cells pretreated with either 15 μM AMTB or vehicle. **B** IC_50_ values of four osteosarcoma cell lines after incubation with AMTB for 48 h. **C** Osteosarcoma cells treated with AMTB exhibited crippled ability of colony formation, and both the amount and the size of the colonies were reduced on a dose-dependent manner. The data was compared by one-way ANOVA with Tukey’s multiple comparison test and shown as mean ± s.d. ***P < 0.001. **D** AMTB suppresses osteosarcoma cell viability in a time- and dose-dependent manner. Osteosarcoma cells were treated with different concentrations of AMTB for indicated time, while cell viability was determined by the MTS assay. The data was compared by two-way ANOVA with Bonferroni test and shown as mean ± s.d. ***P < 0.001. **E**, **F** Osteosarcoma cells were treated with various concentrations of AMTB, and their migration ability was assessed by the wound healing assay and the transwell assay. The data was compared by one-way ANOVA with Tukey’s multiple comparison test and shown as mean ± s.d. ***P < 0.001.

AMTB is a specific antagonist of TRPM8. As shown in Fig. 2A, 143B and U2OS cells pretreated with 15 μM AMTB for 15 min exhibited no response to TRPM8 activator, while TRPM8 activator elicited a significant inward current in the control group. The half inhibitory concentrations (IC50) were then calculated, ranging from 10.49 μM to 13.09 μM, indicating an inhibitory role of AMTB on osteosarcoma cells (Fig. 2B). Meanwhile, further validation of TRPM8 being the target of AMTB using knockdown of the receptor was performed. After knockdown of TRPM8, the IC50 value for the osteosarcoma cells was markedly increased (Fig. S1). The IC50 for hMSC cell was 73.13 μΜ almost five times compared to the osteosarcoma cell lines which showed that the drug was not similarly toxic to normal cells. (Fig. S2). Next, AMTB of gradient doses were used to treat different osteosarcoma cell lines for 72 h. For further exploration of its antiproliferative activity, the MTT assay and the colony formation assay were conducted on U2OS cells and 143B cells. As for the colony formation assay, osteosarcoma cells treated with AMTB exhibited crippled ability of colony formation, and both the amount and the size of the colonies were reduced on a dose-dependent manner (Fig. 2C). During the MTT assay, AMTB exhibited an antiproliferative effect on osteosarcoma cells in both time-dependent and dose-dependent fashion (Fig. 2D). Besides tumor growth, metastasis stands another hallmark in tumor progression. Therefore, transwell assay and wound healing assay were conducted to assess the effect of AMTB on cell migration. Results from both experiments are consistent and collectively indicate a compromised migratory ability of osteosarcoma cells after being treated with AMTB (Fig. 1E, F). Conclusively, the function of TRPM8 is necessary to sustain proliferation and migration for osteosarcoma cells. By disturbing the function of TRPM8, AMTB can suppress the development and the progression of osteosarcoma.

### AMTB induces apoptosis in osteosarcoma cells

**Fig. 3:**
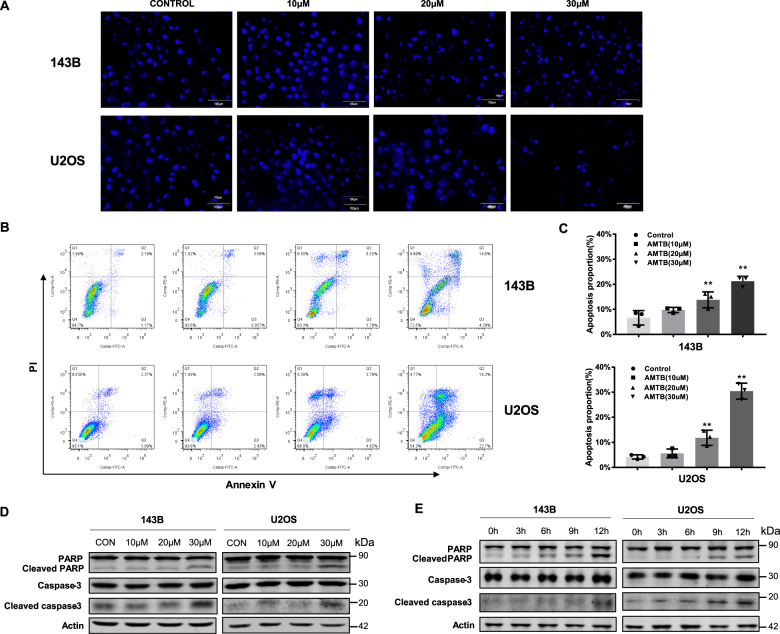
AMTB induces apoptosis in osteosarcoma cells. **A** Hoechst staining revealed typical morphologic changes during apoptosis after treatment with 30 μM AMTB for 24 h. **B**, **C** Annexin V/PI staining revealed a significant increase in apoptosis rate in 143B cells and U2OS cells after incubation with AMTB for 24 h. The data was compared by one-way ANOVA with Tukey’s multiple comparison test and shown as mean ± s.d. **P < 0.01. **D, E** The level of cleaved caspase-3 and cleaved PARP was examined by western blot assay and an increased cleavage of caspase-3 and PARP was observed in a time- and dose-dependent manner. Western blot is representative of three independent western blot with different lysates.

The effect of AMTB on cell apoptosis was further analyzed with Hoechst 33342 staining and flow cytometry to detect the morphologic changes and to analyze the proportion of apoptotic cells. Through Hoechst 33342 staining, we observed more morphologic changes in nuclear chromatin of U2OS cells and 143B cells after treatment with 30 μM AMTB for 24 h. Typical morphologic characteristics of apoptosis, such as reduction in nuclear size, cell pyknosis, and chromatin condensation, were more frequently observed in AMTB-treated cells than in cells treated with vehicle (Fig. 3A). Additionally, Annexin V/PI staining revealed a significant increase in apoptosis rate in 143B cells and U2OS cells after incubation with AMTB for 24 h (Fig. 3B, C). Moreover, we also examined the level of cleaved caspase-3 and cleaved poly (ADP-ribose) polymerase (PARP), 2 prominent characters during cell apoptosis, through western blot assay. And an increased cleavage of these two proteins were observed in both time-dependent and dose-dependent manner, verifying the function of AMTB in inducing cell apoptosis (Fig. 3D, E).

### AMTB exhibits antitumoral function through repressing the TGFβ signaling pathway

To uncover the underlying mechanism by which AMTB exhibits its antitumoral function, we conducted RNA sequencing analysis on U2OS cells treated with either 30 μM AMTB or vehicle.

**Fig. 4:**
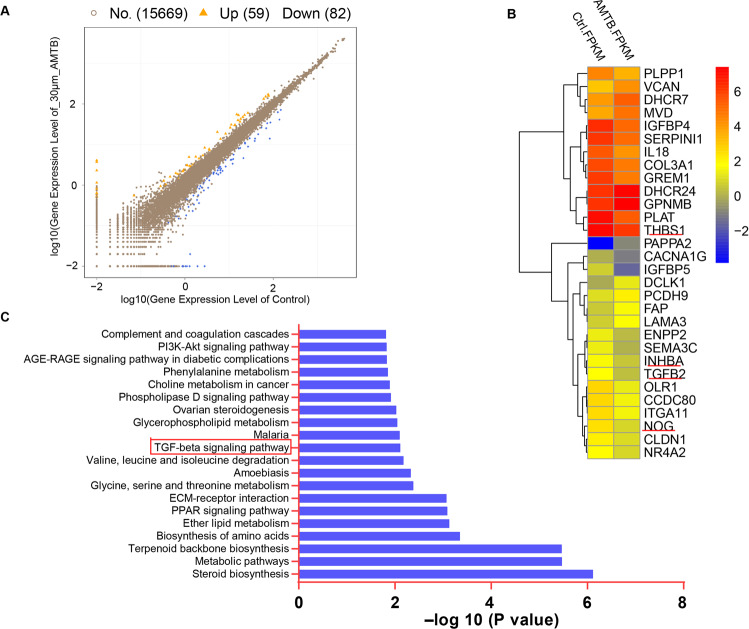
The differentially expressed genes and signal pathway are involved in the treatment of AMTB in osteosarcoma. **A** Differentially expressed genes between AMTB 30 μM treatment and control group, with fold change ≥ 2 and *p* value ≤ 0.05. **B** Heatmap colored according to the expression PFKM value, showing all of the differentially expressed genes involved in proliferation and metastasis. The red represent genes that are upregulated, and the blue represent genes that are downregulated. THBS1, INHBA, TGFB2, and NOG, primary KEGG enrichment members of this pathway, are indicated. **C** KEGG pathway analysis revealed a downregulation of TGFβ signaling after treatment with AMTB 30 μM in osteosarcoma cells.

The differentially expressed genes (DEGs) were identified based on a false discovery rate threshold of 0.05 and fold change of 2. Here, a total of 141 differentially expressed genes were identified, 59 genes up and 82 down (Fig. 4A). We analyzed the corresponding GO terms or KEGG pathways of these features, which are listed in Supplementary Figs. S3 and S4, and 30 genes were picked for cell proliferation or migration (Fig. 4B). Pathway enrichment analysis, an analysis that maps genes to the KEGG pathways, revealed several important signaling pathways including the TGFβ pathway and PI3K pathway had significantly downregulated genes (Fig. 4C). However, our results indicated that the AMTB could not inhibit the phosphorylation of PI3K and AKT in a dose-dependent manner (Fig. S5). The most statistically significant differentially expressed genes of the TGFβsignaling pathway components were shown in the heatmap, including TGFB2, THBS1, NOG, and INHBA (Fig. 4B). We further analyzed the protein–protein interaction networks using data from the STRING database. The results showed that the DEGs formed a densely connected network (Fig. S6), suggesting that these genes work as a functional module at the protein level.

**Fig. 5:**
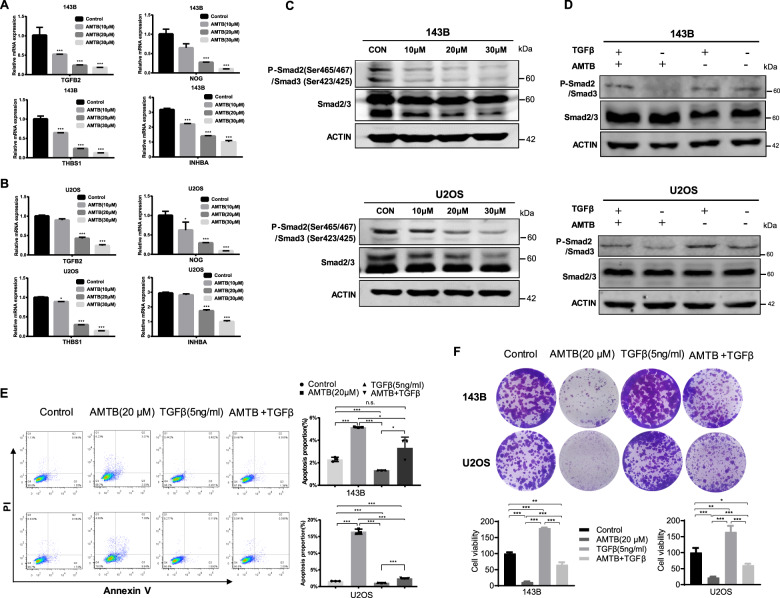
AMTB exhibits antitumoral function through repressing the TGFβ signaling pathway. **A**, **B** RT-qPCR was conducted to detect the transcriptional level of TGFβ and relevant targets of TGFβ signaling after treatment with various concentrations of AMTB. **C** Western blot assay on P-Smad2 and P-Smad3 indicated that AMTB can inhibit the phosphorylation of Smad2 and Smad3 in a dose-dependent manner. Western Blot is representative of three independent western blot with different lysates. **D** By adding TGFβ (5 ng/ml) to AMTB-treated osteosarcoma cells, the phosphorylation level of Smad2 and Smad3 was rescued to basal level. Western blot is representative of three independent western blot with different lysates. **E**, **F** Annexin V/PI staining revealed a variation in apoptosis rate in 143B cells and U2OS cells after different treatment, and their proliferation ability was assessed by the colony formation assay. The data was compared by one-way ANOVA with Tukey’s multiple comparison test and shown as mean ± s.d. *P < 0.05, **P < 0.01, ***P < 0.001.

Furthermore, we carried out RT-qPCR to test the transcriptional level of TGFβ and several enrichment gene targets (KEGG analysis) of TGFβ signaling in different treatment groups, and the results were consistent with our previous speculations (Figs. 4B and 5A, B). Since the phosphorylation of Smad2 and Smad3 plays a pivotal role in TGFβ signaling, we tested the phosphorylation level of Smad2 and Smad3 after treating osteosarcoma cells with gradient concentrations of AMTB. As the results indicated, AMTB can inhibit the phosphorylation of Smad2 and Smad3 in a dose-dependent manner (Fig. 5C). Finally, we added TGFβ (5 ng/ml) to AMTB-treated osteosarcoma cells and observed that the phosphorylation level of Smad2 and Smad3 was rescued to basal level (Fig. 5D). To further verify the effects of the proliferation and apoptosis on the TGF-β pathway components whether showed independent effects on the osteosarcoma cell, we have done the Annexin V/PI staining and relevant colony formation assay to reveal the variation in apoptosis and proliferation in osteosarcoma cells. Figure 5E, F indicated that AMTB can inhibit the proliferation of osteosarcoma cells and increased the apoptosis rate while the third group (TGFβ (5 ng/ml)) showed adverse results compared with the former paired group. Furthermore, in the fourth group(AMTB + TGFβ), the above phenotype was rescued. Therefore, the above results corroborated that AMTB exhibits its antitumoral effect through repressing the activation of TGFβ signaling and activating the pathway would rescue that phenotype.

### AMTB can increase the sensitivity of osteosarcoma cells to cisplatin in vitro and in vivo

**Fig. 6:**
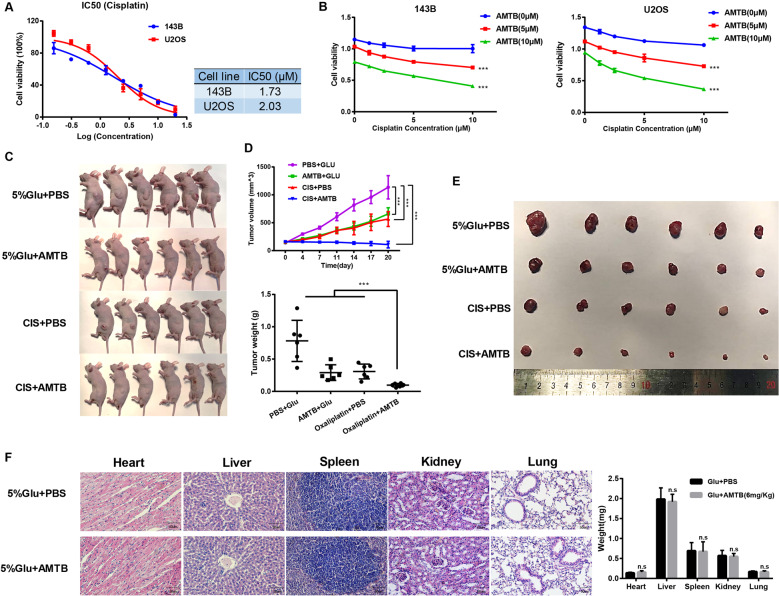
AMTB can increase the sensitivity of osteosarcoma cells to cisplatin in vitro and in vivo. **A** IC50 values of U2OS cells and 143B cells after incubation with cisplatin for 48 h. **B** Combination of various concentrations of AMTB and cisplatin were used to treat osteosarcoma cells, and the results indicated that AMTB combined with low dosage of cisplatin exhibited stronger suppressive effect compared with high dosage of cisplatin alone. The data was compared by two-way ANOVA with Bonferroni test and shown as mean ± s.d. ***P < 0.001. **C** Preclinical experimental model of osteosarcoma induced by subcutaneous injection of osteosarcoma cells into the back of the mice. *n* = 6. **D** Tumor volume was recorded every two days after indicated treatment, and tumors were removed and weighted at the termination of the study. The data was compared by two-way ANOVA with Bonferroni test and shown as mean ± s.d. ***P < 0.001. **E** The isolated xenograft tumors from treated or control group were shown. *n* = 6. **F** Vital organs amongst different treatment groups were weighted and showed no significant difference. H&E staining of vital organs amongst different treatment groups indicated that no histologic changes were induced by ATMB treatment. The data was compared by one-way ANOVA with Tukey’s multiple comparison test and shown as mean ± s.d. **P < 0.01, ***P < 0.001.

Cisplatin is one of the most used cytotoxic chemotherapeutic regimens in treating osteosarcoma. The IC50 value of cisplatin was 1.73 μM for 143B cells and 2.03 μM for U2OS cells (Fig. 6A). We then treated osteosarcoma cells using various concentrations of AMTB and cisplatin, and the results indicated that AMTB combined with low dosage of cisplatin exhibited stronger suppressive effect compared with high dosage of cisplatin alone (Fig. 6B). For further investigation, a preclinical experimental model of osteosarcoma was developed, induced by subcutaneous injection of osteosarcoma cells. Approximately 5 × 10^6^ U2OS cells were subcutaneously injected into the back of 6-week female nude mice. After 10 days’ observation, 24 mice bearing similar volume of tumors were randomly attributed into four treatment groups: a control group, a AMTB-alone treatment group, a cisplatin-alone treatment and a group treated with a combination of cisplatin and AMTB. Mice in different groups were treated accordingly on a once every 2 days’ basis and were treated for a total of 3 weeks. At the termination of the study, a significant reduction of tumor size was observed. Our in vivo data indicated a robust combination effect of AMTB together with the cisplatin when compared with the efficacy of either treatment alone (Fig. 6D). While cisplatin or AMTB as single agents modestly inhibited tumor growth, the combination was efficiently capable of arresting tumor cell growth in the xenograft tumor. In the meanwhile, the average tumor weight was 0.31 ± 0.12 g for mice treated with cisplatin and 0.10 ± 0.03 g for mice treated with cisplatin and AMTB (*p* = 0.002) (Fig. 6C–E). On the contrary, the average weight of vital organs amongst different treatment groups were not significantly different, and no obvious histologic changes were observed as detected by the hematoxylin and eosin (H&E) staining (Fig. 6F).

## Discussion

First identified during a screening for upregulated genes in prostate cancer, TRPM8 is subsequently found to be overexpressed in a wide range of cancers such as breast cancer and pancreatic adenocarcinoma [11, 27, 28]. Such increased expression revealed by either RT-qPCR or IHC staining are found correlated with disease progression and patients’ prognosis [12, 29]. In the present study, we demonstrated an upregulation of TRPM8 in osteosarcoma cell lines compared to MSC. In addition, through analyzation of clinical samples, we also observed a differential expression of TRPM8 between osteosarcoma and adjacent non-tumoral tissues. Notably, strong staining for TRPM8 in clinical samples is found relevant with patients’ overall survival, verifying its potential role as a molecular marker and prognostic indicator in osteosarcoma (OS: 40.0 ± 5.8 months vs 21.6 ± 2.6 months, *p* = 0.008).

Clinical studies revealing the overexpression of TRPM8 in various tumors give rise to the hypothesis that TRPM8 may be implicated in tumorigenesis and tumor progression. Based on this speculation, a series of studies were carried out, trying to investigate the role of TRPM8 in tumor proliferation and migration. Small interfering RNA directed against TRPM8 is found to be able to interrupt cell cycle progression, suppress migration, decrease basal autophagy and enhance the effect of cytotoxic chemotherapeutic agents [11, 17, 30, 31]. In the meanwhile, in vitro interfering of the normal function of TRPM8 proves to have minor side effect on immortalized normal cells, making TRPM8 a valuable therapeutic target in tumor treatment [17, 28]. Unlike many of the ubiquitously expressed anticancer drug targets (such as the regulators of the cell cycle), therapies based on targets with limited tissue distributions are less likely to be associated with generalized toxicity [32]. In this context, several antagonists against TRPM8, such as BCTC, sesamin, and cannabigerol, were tested in various cancers, and proved to have an antitumoral effect [16, 33, 34]. AMTB, as a specific TRPM8 antagonist, has already been known to be able to attenuate TRPM8-mediated cold hyperalgesia and nociception [14, 35]. However, its growth-inhibitory effect has only been tested on breast cancer (MDA-MB-231) in one study, which failed to specify the function of AMTB or provide any underlying molecular mechanisms [36]. Our study demonstrates for the first time that AMTB has an antitumoral function against osteosarcoma and can increase the effect of cisplatin in vitro and in vivo.

Cisplatin is one of the most common chemotherapeutic regimens in the treatment of osteosarcoma. It is known to be able to induce peripheral neuropathy which can be aggravated by exposure to cold [37]. Biologically, TRPM8 is implicated in cold sensation and nociception. Recent studies demonstrate that TRPM8 can promote local vasodilation which can aggravate platinum-induced peripheral neuropathy [38]. Additionally, TRPM8 is also implicated in platinum-induced cold allodynia and cold dysesthesia, which can be attenuated by TRPM8 antagonist [10, 39]. Given the previous findings, AMTB may also plays a role in alleviating platinum-induced peripheral neuropathy through antagonizing TRPM8. In our study, we further proved the antitumoral ability of AMTB in vivo, and proved that AMTB combined with low dosage of cisplatin can achieve better antitumoral effect than high dose of cisplatin alone, suggesting the possible value of combining AMTB and cisplatin in the treatment of osteosarcoma. In the meanwhile, the feasibility of targeting Ca^2+^ channels has already been highlighted by clinical experience of voltage gated Ca^2+^ channel blockers for the treatment of hypertension, and also by the viable phenotype of TRPM8−/− mice [40].

Through further investigations, we proved that AMTB exhibits its antitumoral effect by suppressing the phosphorylation of Smad2 and Smad3, thus the activation of TGFβ signaling. Perturbations of TGFβ signaling are implicated in tumorigenesis and tumor progression [20]. On one hand, TGFβ serves as an autocrine growth factor, which favors osteosarcoma progression through the induction of PDGF [41]. On the other hand, TGFβ also has crucial roles in host-tumor interactions. Evidence shows that osteosarcoma cells can release TGFβ-rich extracellular vesicles, which can induce the prometastatic IL-6 production by MSC [42]. In the meanwhile, TGFβ promotes local angiogenesis and bone remodeling, and is implicated in tumor immunosurveillance, exerting a pro-tumorigenic function [41, 43, 44]. Ca^2+^ homeostasis may play a crucial role in TRPM8-mediated suppression of TGFβ signaling during AMTB treatment. In previous studies, nuclear factor of activated T-cells 1 (NFAT1), acting as a Ca^2+^-dependent transcription factor, was found to be able to promote the expression and secretion of TGFβ to promote tumor metastasis [45].

## Conclusions

In summary, our study revealed an upregulation of TRPM8 in osteosarcoma, and the expression level of TRPM8 is negatively related to patients’ prognosis. In addition, our results demonstrate for the first time that AMTB has an antitumoral function against osteosarcoma and can increase the effect of cisplatin both in vitro and in vivo. Finally, we proved that AMTB exhibits its antitumoral effect by suppressing the phosphorylation of Smad2 and Smad3, thus the activation of TGFβ signaling.

## Supplementary information

Authors’ contributions

supplementary legends

Figure S1

Figure S2

Figure S3

Figure S4

Figure S5

Figure S6

aj-checklist

## Data Availability

The data used or analyzed during this study are included in this article and available from the corresponding author upon reasonable request.
