# Supplementary material for: AMTB, a TRPM8 antagonist, suppresses growth and metastasis of osteosarcoma through repressing the TGFβ signaling pathway
Source: Cell Death Dis. 2022 Mar 31;13(3):288. doi: 10.1038/s41419-022-04744-6 (PMC8971393; doi:10.1038/s41419-022-04744-6)
Supplement: Supplementary file 1 — Authors’ contributions [file 41419_2022_4744_MOESM1_ESM.docx]

**Authors’ contributions**

XJR, ZD and LK designed the study. LYJ, LA, LL performed most of the experiments. YB, SSH and ZEH performed part of the experiments. CH, SPL, XQY, MXY, LTL and LFR analyzed and interpreted the data. LYJ, LK and LA wrote and revised the manuscript. All authors read and approved the final manuscript.
