# Supplementary material for: AMTB, a TRPM8 antagonist, suppresses growth and metastasis of osteosarcoma through repressing the TGFβ signaling pathway
Source: Cell Death Dis. 2022 Mar 31;13(3):288. doi: 10.1038/s41419-022-04744-6 (PMC8971393; doi:10.1038/s41419-022-04744-6)
Supplement: Supplementary file 2 — supplementary legends [file 41419_2022_4744_MOESM2_ESM.docx]

**Fig. S1 A and B** IC50 and qPCR for each cell line including 143B, U2OS and their knockdown cell line.

**Fig. S2** IC50 for hMSC cell line.

**Fig. S3** GO functional enrichment analysis revealed all of the differentially expressed genes involved in proliferation and metastasis after treatment with AMTB 30μM in osteosarcoma cells.

**Fig. S4** KEGG pathway analysis revealed a downregulation of top 20 signaling after treatment with AMTB 30μM in osteosarcoma cells.

**Fig. S5** Western blot assay on p-PI3K and p-AKT indicated that AMTB could not inhibit the phosphorylation of PI3K and AKT in a dose-dependent manner.

**Fig. S6** Protein–protein interaction networks, functional modules consisting of the enriched 141 differentially expressed downstream genes.
