# Supplementary figures and images for: AMTB, a TRPM8 antagonist, suppresses growth and metastasis of osteosarcoma through repressing the TGFβ signaling pathway
Source: Cell Death Dis. 2022 Mar 31;13(3):288. doi: 10.1038/s41419-022-04744-6 (PMC8971393; doi:10.1038/s41419-022-04744-6)

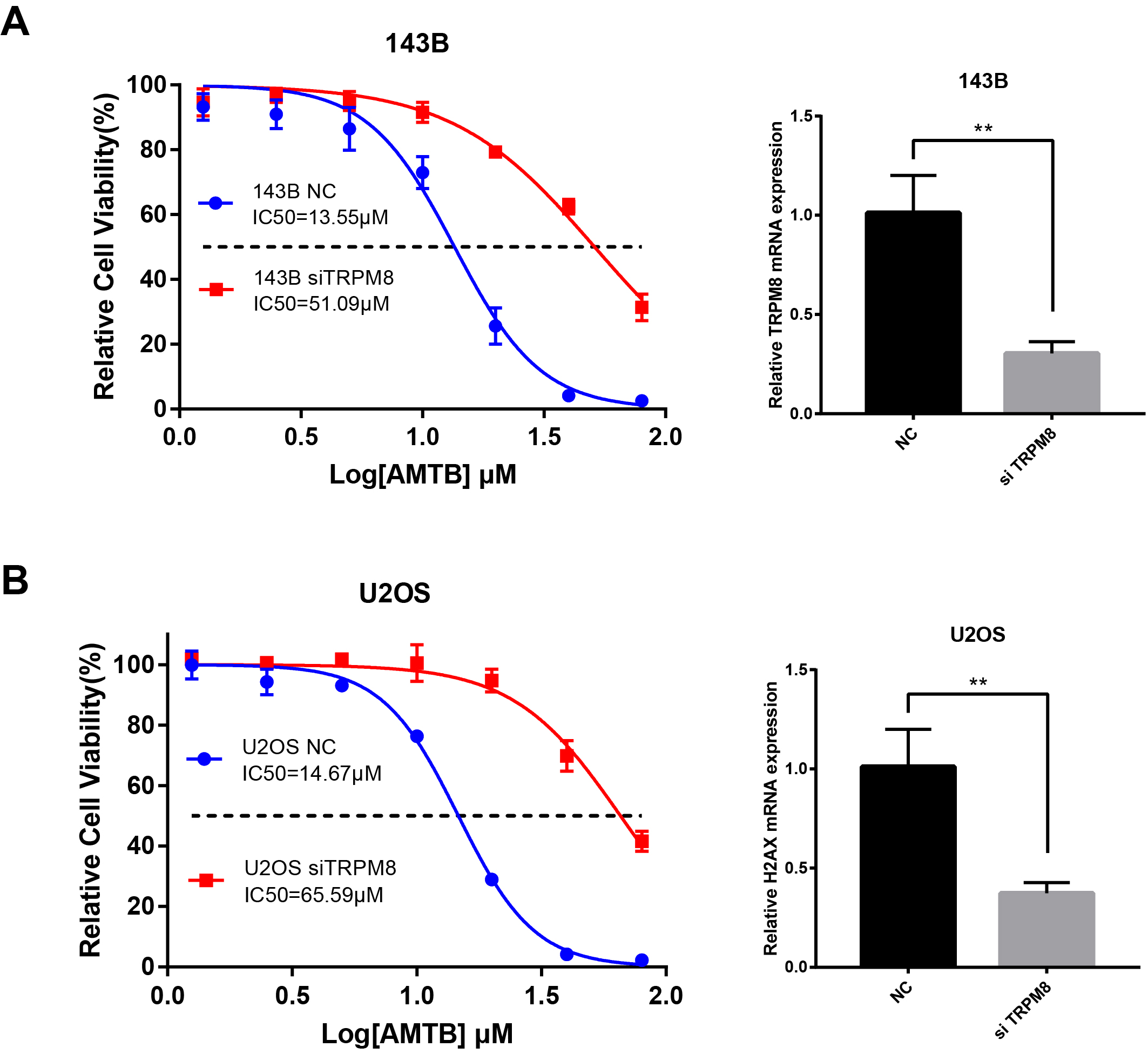

Supplement: Supplementary file 3 — Figure S1 [file 41419_2022_4744_MOESM3_ESM.jpg]

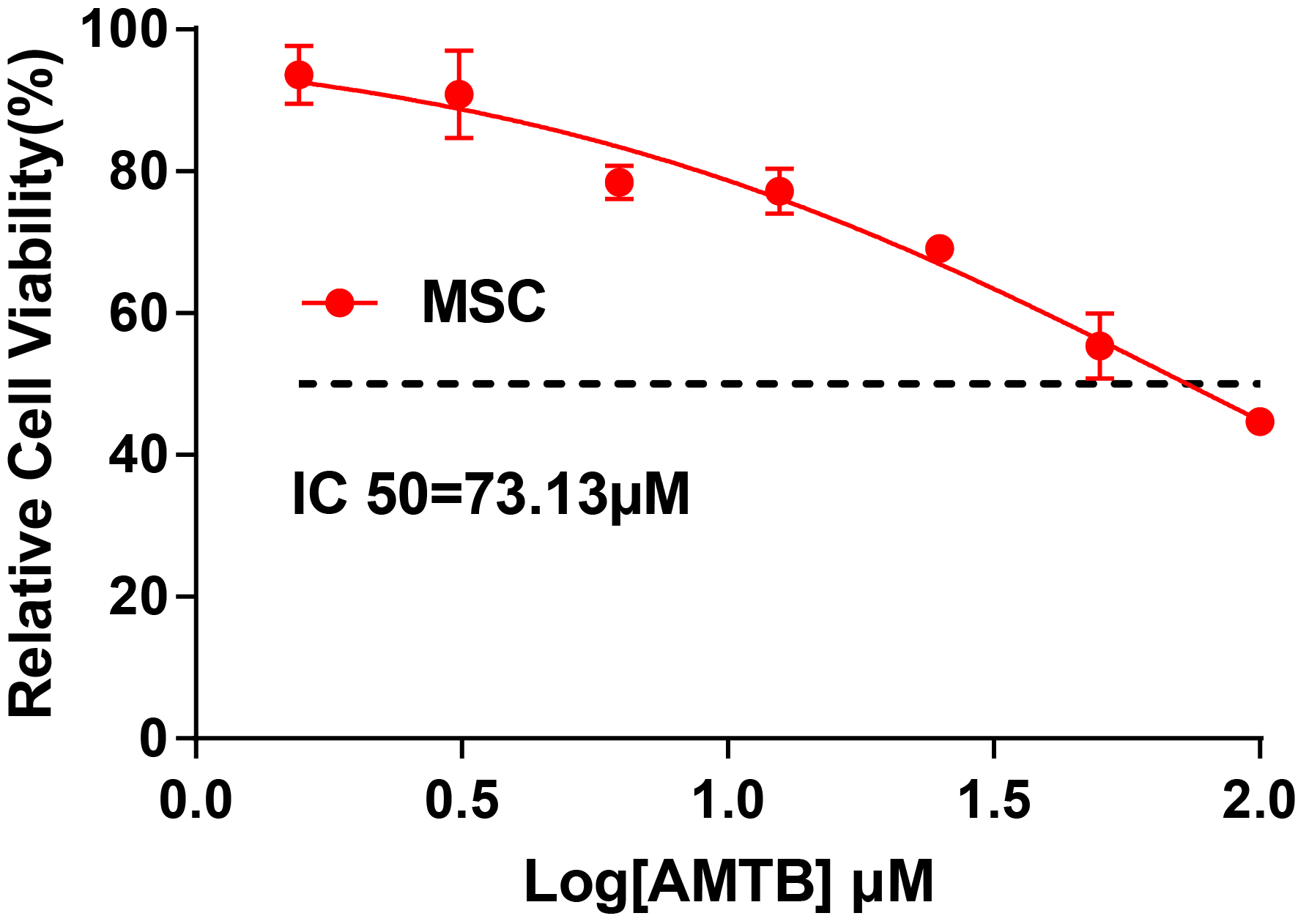

Supplement: Supplementary file 4 — Figure S2 [file 41419_2022_4744_MOESM4_ESM.jpg]

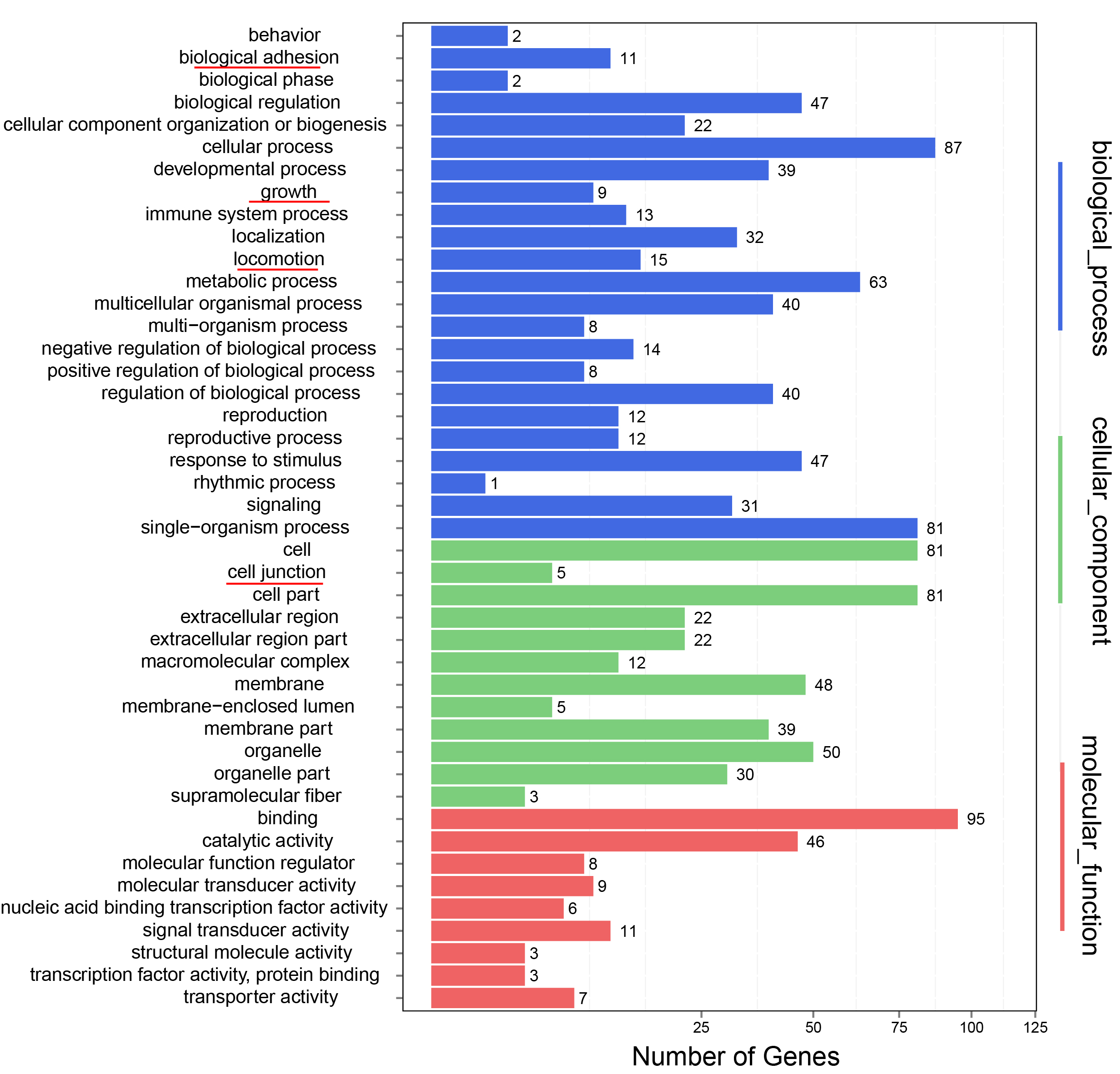

Supplement: Supplementary file 5 — Figure S3 [file 41419_2022_4744_MOESM5_ESM.jpg]

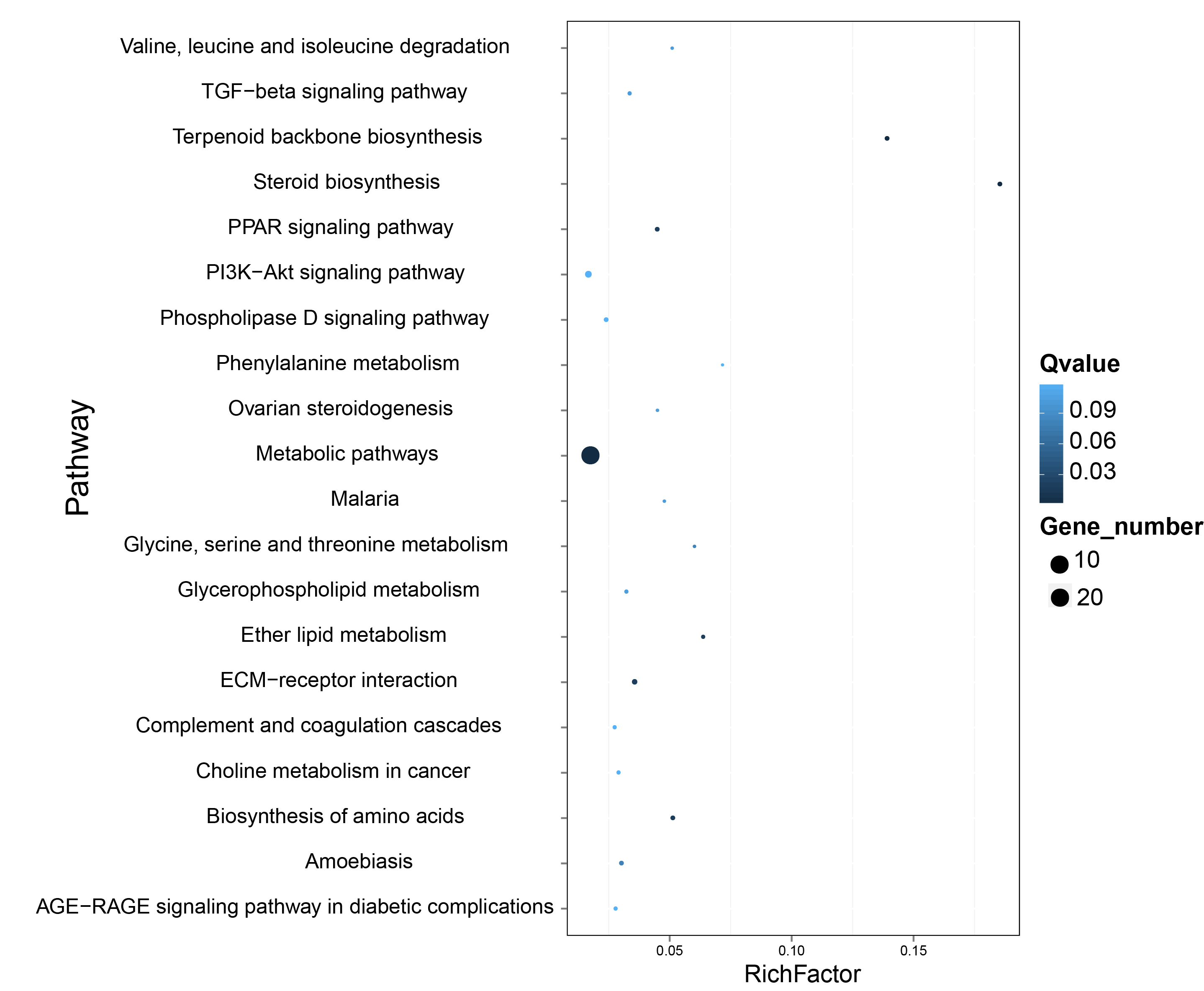

Supplement: Supplementary file 6 — Figure S4 [file 41419_2022_4744_MOESM6_ESM.jpg]

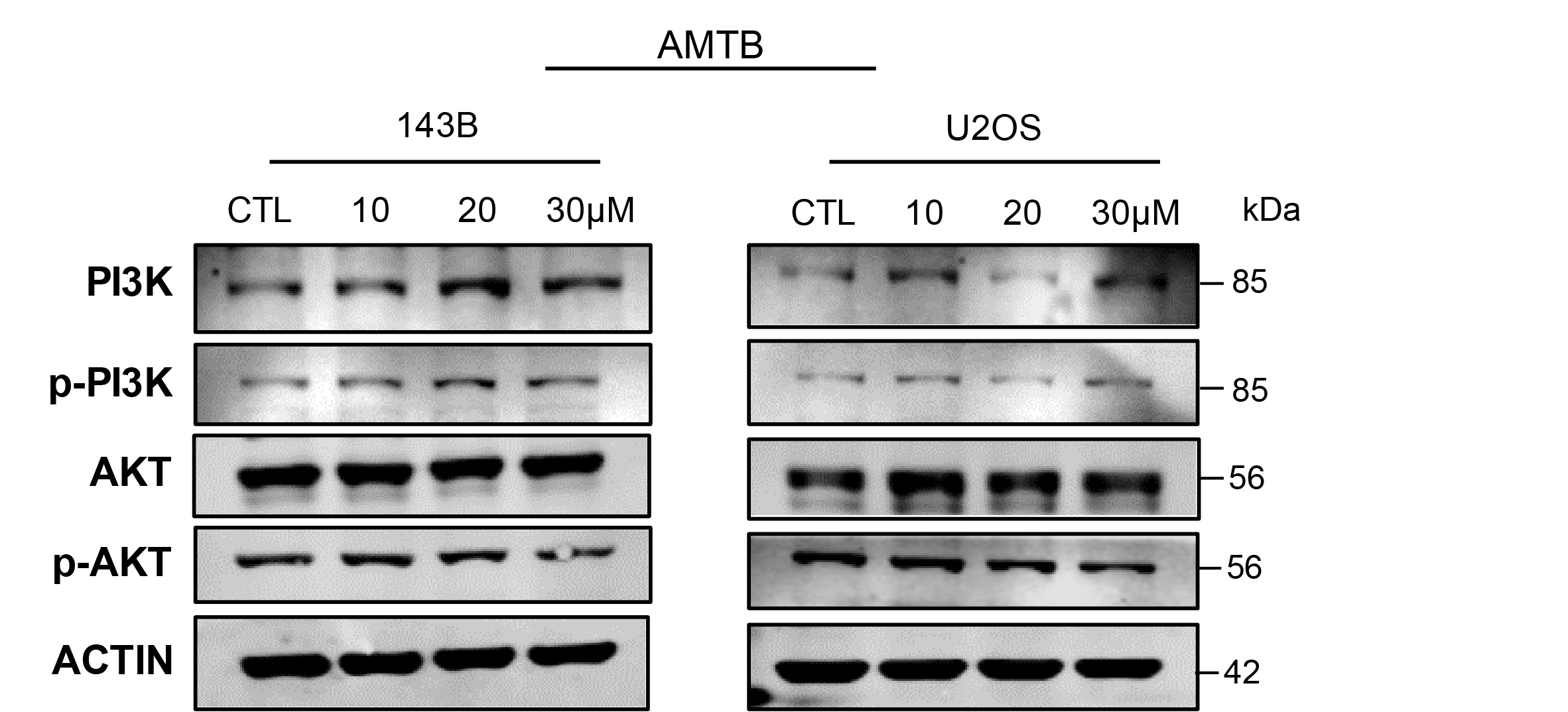

Supplement: Supplementary file 7 — Figure S5 [file 41419_2022_4744_MOESM7_ESM.jpg]

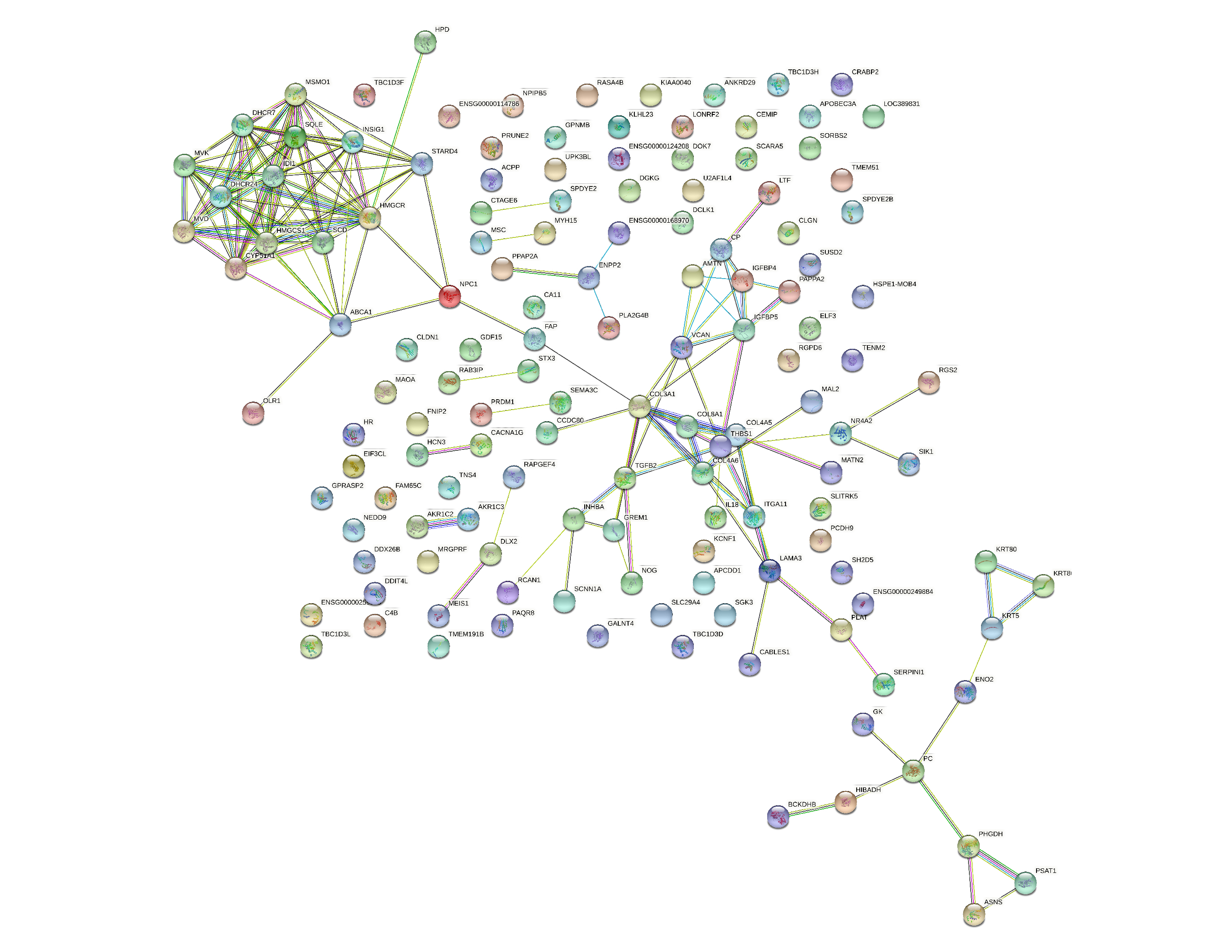

Supplement: Supplementary file 8 — Figure S6 [file 41419_2022_4744_MOESM8_ESM.jpg]
